# High Prevalence of Multidrug-Resistant Tuberculosis, Swaziland, 2009–2010

**DOI:** 10.3201/eid1801.110850

**Published:** 2012-01

**Authors:** Elisabeth Sanchez-Padilla, Themba Dlamini, Alexandra Ascorra, Sabine Rüsch-Gerdes, Zerihun Demissie Tefera, Philippe Calain, Roberto de la Tour, Frauke Jochims, Elvira Richter, Maryline Bonnet

**Affiliations:** Epicentre, Paris, France (E. Sanchez-Padilla, A. Ascorra, M. Bonnet);; National Tuberculosis Control Programme, Mbabane, Swaziland (T. Dlamini);; National Reference Center for Mycobacteria, Borstel, Germany (S. Rüsch-Gerdes, E. Richter);; Médecins Sans Frontières, Geneva, Switzerland (Z.D. Tefera, P. Calain, R. de la Tour, F. Jochims)

**Keywords:** bacteria, Mycobacterium tuberculosis, tuberculosis, MDR, drug resistance, multidrug resistance, Africa, HIV, cross-sectional studies, Swaziland, TB, antimicrobial resistance

## Abstract

In Africa, although emergence of multidrug-resistant (MDR) tuberculosis (TB) represents a serious threat in countries severely affected by the HIV epidemic, most countries lack drug-resistant TB data. This finding was particularly true in the Kingdom of Swaziland, which has the world’s highest HIV and TB prevalences. Therefore, we conducted a national survey in 2009–2010 to measure prevalence of drug-resistant TB. Of 988 patients screened, 420 new case-patients and 420 previously treated case-patients met the study criteria. Among culture-positive patients, 15.3% new case-patients and 49.5% previously treated case-patients harbored drug-resistant strains. MDR TB prevalence was 7.7% and 33.8% among new case-patients and previously treated case-patients, respectively. HIV infection and past TB treatment were independently associated with MDR TB. The findings assert the need for wide-scale intervention in resource-limited contexts such as Swaziland, where diagnostic and treatment facilities and health personnel are lacking.

## Abstract 2

One third of previously treated patients had MDR TB.

Despite efforts to control the tuberculosis (TB) epidemic, there were an estimated 9.4 million incident cases of TB worldwide in 2009 (*1*). The HIV epidemic and the emergence of anti-TB drug resistance represent serious threats for achieving the Stop TB Partnership’s goal of eliminating TB as a public health problem by 2050 (*2*). HIV co-infected patients are more likely to show development of active TB. Even though antiretroviral therapy for HIV reduces this risk, TB remains 5× more frequent in persons living with HIV/AIDS (*3*). Indeed, 30% of the patients in whom TB was diagnosed in 2008 worldwide were in Africa, possibly because of the HIV epidemic affecting the continent (*1*).

Patients infected with a *Mycobacterium* spp. strain resistant to rifampin and isoniazid, which defines a multidrug-resistant (MDR) TB strain, do not respond to World Health Organization (WHO) standardized directly observed short-course chemotherapy and require longer, more toxic, and more expensive treatment. Timely identification of patients with MDR TB enables rapid initiation of adequate treatment, thus preventing the patient from spreading the disease and from acquiring further resistance.

Ideally, routine drug susceptibility testing (DST) should be conducted before initiation of treatment in all patients with TB, but this is not achievable in most high-prevalence countries because of poor access to bacterial culture and DST tools. For the same reasons, in most countries with a high prevalence of TB, no surveillance of anti-TB drug resistance is conducted. Periodic surveys of a representative sample of patients with TB often constitute the only available sources of information on the prevalence of drug resistance (*4*). In the last WHO report on resistance to anti-TB drugs, data from periodic surveys with relatively recent data were available for only 21 of 46 African countries (*5*).

The Kingdom of Swaziland, in southern Africa, is the country with the world’s highest HIV prevalence (26% among adults in 2007) and TB incidence rate per capita (1,257 cases per 100,000 population in 2009) (*6**,**7*). In 2007, in collaboration with the Ministry of Health and Social Welfare of Swaziland, Médecins Sans Frontières started an integrated HIV/TB project in Shiselweni in southern Swaziland.

In Swaziland, the last national anti-TB drug resistance survey had been conducted in 1995 and reported an MDR TB prevalence of 0.9% among new TB case-patients and 9.1% among previously treated case-patients (*5*). Several factors could have potentially resulted in an increase of MDR TB in the country in recent years and in emergence of extensively drug-resistant (XDR) TB, which is defined as MDR TB resistant to >1 injectable second-line drug and any fluoroquinolone. The National TB Control Programme in Swaziland had reported relatively poor TB treatment success rates (68% and 48% for new and retreatment smear-positive TB case-patients, respectively, in 2008), with high failure rates (7% in new case-patients and 11% in retreated case-patients) (*1*). Additionally, Swaziland borders the province of KwaZulu-Natal in South Africa, where an outbreak of XDR TB was reported in 2005 among HIV co-infected patients (*8*); many citizens of Swaziland regularly cross the border to work in South African mines. In 2007, the Ministry of Health and Social Welfare conducted a rapid survey among high-risk patients to detect the occurrence of XDR TB and reported 4 patients with XDR TB and an 18.5% MDR TB prevalence among previously treated case-patients (*9*). These findings justified the need for a new national anti-TB drug resistance survey that measured the current prevalence of MDR TB among new and previously treated patients with TB in Swaziland.

## Methods

### Design and Study Population

**Figure 1:**
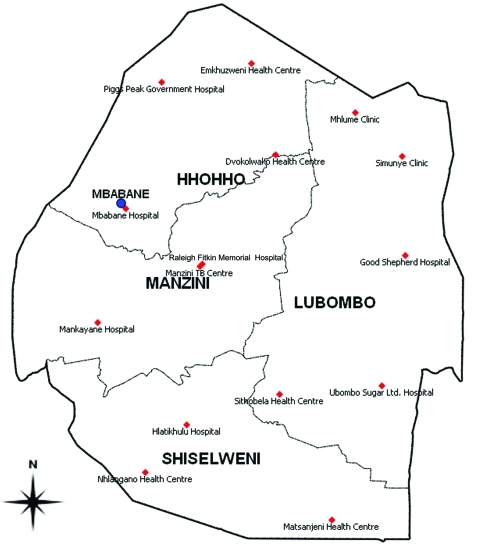
Tuberculosis diagnosis facilities (red diamonds), Swaziland.

A cross-sectional survey was designed based on the most recent WHO guidelines for surveillance of drug resistance in TB (*4*). The 15 TB diagnosis centers of the 4 regions of Swaziland participated in the study. These included the Manzini TB center, 7 general hospitals, 5 health centers, and 2 clinics (Figure 1).

Consecutive smear-positive patients >14 years of age who were given a new diagnosis of TB were invited to participate in the survey. Patients were considered as new case-patients (NCs) if they had never received treatment for TB or had taken anti-TB drugs for <1 month in the past or as previously treated case-patients (PTCs) if they had ever received anti-TB drugs for >1 month. PTCs included patients who returned after defaulting treatment, experienced TB relapses, or had TB treatment failure according to WHO case definitions (*10*). To be consistent with the guidelines of the National TB Control Programme, a treatment failure case-patient was defined as a person who remained smear positive after 3 months of treatment and not 5 months as recommended by WHO (*10*).

### Procedures

Data from screened patients were collected on a standardized form that included demographics (e.g., age, sex, region of residence), duration of illness, smear microscopy results, history of TB treatment (number of previous anti-TB treatment courses and outcomes), and HIV status. A 1-day training program was organized for all personnel participating in administering the survey during the month before the beginning of the study. Patient recruitment started after a pilot phase of 2 weeks where study procedures, including the transport of specimens, were assessed. Weekly site-monitoring visits were organized to support each site during patient recruitment.

Sputum smear examination was performed at the TB diagnostic centers on 2 sputum specimens from each patient, collected during 2 consecutive days, by using the hot Ziehl-Neelsen method. A smear-positive case-patient was defined by >1 positive smear result with >1 acid-fast bacilli per 100 high-power microscopic fields, as recommended by WHO (*11*). Smear-positive case-patients were asked to produce an extra on-the-spot sputum sample, which was stored in a refrigerator (4°C) until shipment to the Supranational Reference Centre for Mycobacteria in Borstel, Germany, for culture and DST. Specimens were shipped weekly.

For cultures, samples were placed into liquid medium by using the BACTEC MGIT 960 system (Becton Dickinson, Franklin Lakes, NJ, USA) and on 2 solid media (Löwenstein-Jensen and Stonebrink). *M. tuberculosis* was identified by using the GenoType MTBC test (HAIN Lifescience GmbH, Nehren, Germany). For mycobacteria other than *M. tuberculosis*, the GenoType Mycobacterium CM/AS test (HAIN Lifescience GmbH) or DNA sequencing was performed. DST of first-line anti-TB drugs (i.e., rifampin, isoniazid, streptomycin, and ethambutol) was performed on all positive culture samples. Susceptibility to pyrazinamide was tested on strains resistant to rifampin, isoniazid, ethambutol, or streptomycin. DST of second-line anti-TB drugs, which are defined as a group of drugs active against TB used in case of resistance or intolerance to first-line drugs, was performed on strains resistant to rifampin, isoniazid, or both. Second-line drugs tested were amikacin, capreomycin, ofloxacin, 4-aminosalicylic acid, and ethionamide. Moxifloxacin susceptibility was tested in case of ofloxacin resistance. The indirect proportion method on Löwenstein-Jensen medium was used except for pyrazinamide, ethionamide, and moxifloxacin, which were tested on special acid media in the MGIT 960 (Becton Dickinson).

Free access to voluntary counseling and testing for HIV were offered to any patient given a new diagnosis of TB at each study site. According to national guidelines, positive results for 2 rapid HIV tests were required to define a patient as HIV positive. In the event of discordant results between the 2 tests, DNA PCR by using dried blood spot was performed at the National Reference Laboratory.

### Sample Size and Statistical Analysis

Independent sample sizes were calculated for NCs and PTCs on the basis of the expected prevalence of rifampin resistance per group (5% for NCs and 15% for PTCs), maximal acceptable absolute error of 2.5% for NCs and 4.0% for PTCs, and 95% CI. Sample sizes were increased by 20% to account for expected losses (e.g., contaminated samples, nongrowing cultures, missing DST results).

Data were entered into EpiData version 3.1 software (EpiData Association, Odense, Denmark) and cleaned and analyzed with Stata for Windows version 10.1 (StataCorp LP, College Station, TX, USA). Distributions of categorical variables between 2 groups were compared by using the Fisher exact test. Comparisons of continuous variables were performed by using a 2-sample *t*-test when the variable showed a normal distribution and with a non-parametric Wilcoxon rank-sum test otherwise. Prevalence ratios and 95% CIs were calculated to measure the degree of association between independent variables and MDR TB through generalized linear models for the binomial family. We used an α error of 5% for all statistical tests.

Data quality was assessed through double entry of 5% of the case report forms and regular cross checks between case report forms and data entered in the data. Identified errors were removed before analysis. Missing data was not imputed or replaced.

### Ethical Approval

The study was approved by the Ministry of Health and Social Welfare Scientific Ethical Committee of Swaziland and the Ethics Review Board of Médecins Sans Frontières. Written informed consent to participate to the survey was obtained from patients or from parents or caregivers for adolescents.

## Results

**Figure 2:**
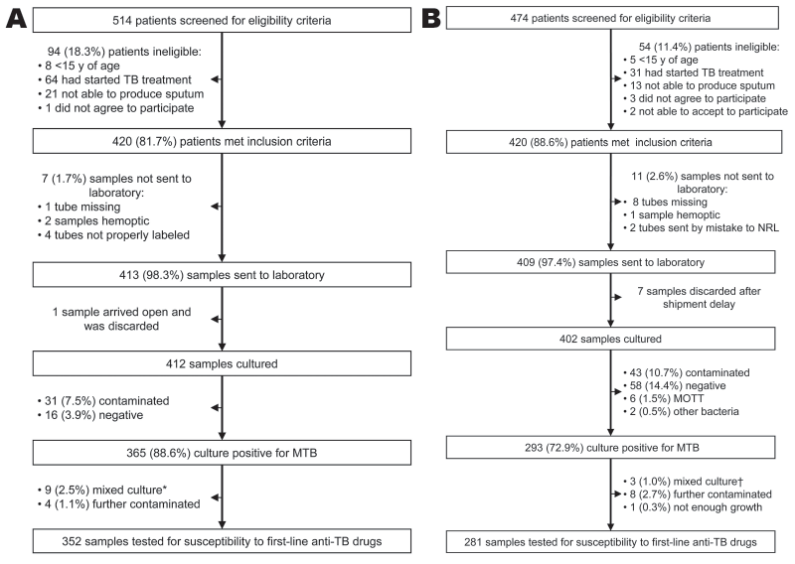
Study profile, national drug-resistant tuberculosis (TB) survey, Swaziland, 2009–2010. A) New patients; B) previously treated patients. NRL, National Reference Laboratory; MTB, *Mycobacterium tuberculosis*; MOTT, mycobacteria other than tuberculosis. *MTB + MOTT. †1 MTB + MOTT, 2 MTB + other bacteria (nonmycobacteria).

Patient recruitment started in May 2009 and was completed in July 2009 for NCs and February 2010 for PTCs. Of 988 screened patients, 840 (85%) met the study inclusion criteria (Figure 2). During the survey period, the national TB program registered 1,175 TB smear-positive case-patients (618 NCs and 557 PTCs). Therefore, 84% of all registered patients were screened for the survey. Reasons given by the health personnel for not screening were heavy workload in the health center and staff turnover. No significant differences in age and sex between screened and nonscreened patients were observed.

Of 822 samples shipped to the mycobacteriology laboratory in Germany, 814 were cultured (99%). Of these samples, 74 (9.1%) were negative and 74 (9.1%) were contaminated. Half the negative culture results (n = 37) were from the PTC subgroup of treatment failure patients.

Fifteen mycobacteria other than *M. tuberculosis* were isolated: *M. avium* (n = 6), *M. avium* complex (n = 5), *M. fortuitum* (n = 2), and *M. kansaii* (n = 2). In 13 cultures, >1 type of mycobacteria grew.

For baseline characteristics of the included patients (Table 1), overall male-to-female ratio was 0.9 and median age was 33 years (interquartile range 27–41 years). The patients were equally distributed among the 4 regions of Swaziland. Regarding socioeconomic aspects, 80.6% of the patients had either not attended school or had not reached high school, and 68.8% had no permanent job.

**Table 1:** Baseline characteristics of case-patients in a study of MDR TB, Swaziland, 2009–2010*

Characteristic	New, n = 420	Previously treated, n = 420	Total, n = 840
Median age, y	32 (IQR 26–40)	33 (IQR 28–43)	33 (IQR 27–41)
Sex			
M	206 (49.0)	196 (46.7)	402 (47.9)
F	214 (51.0)	224 (53.3)	438 (52.1)
Region of residence			
Shiselweni	106 (25.2)	96 (22.9)	202 (24.0)
Manzini	106 (25.2)	119 (28.3)	225 (26.8)
HhoHho	120 (28.6)	109 (26.0)	229 (27.3)
Lubombo	86 (20.5)	96 (22.9)	182 (21.7)
Unknown	2 (0.5)	0	2 (0.2)
Education			
None	97 (23.1)	75 (17.9)	172 (20.5)
Primary school	144 (34.3)	145 (34.5)	289 (34.4)
Secondary school	104 (24.8)	112 (26.7)	216 (25.7)
High school	62 (14.8)	56 (13.3)	118 (14.0)
Tertiary	10 (2.4)	19 (4.5)	29 (3.5)
Unknown	3 (0.7)	13 (3.1)	16 (1.9)
Employment (permanent job)			
No	285 (67.9)	293 (69.8)	578 (68.8)
Yes	127 (30.2)	115 (27.4)	242 (28.8)
Unknown	8 (1.9)	12 (2.9)	20 (2.4)
HIV-positive status known	279/316 (77.3)	327/397 (82.4)	606/758 (79.9)
Previous anti-TB treatment courses			
1		352 (83.8)	
2		39 (9.3)	
>2		14 (3.3)	
Unknown		15 (3.6)	
Previous TB treatment regimen			
Category I		349 (83.1)	
Category II		60 (14.3)	
MDR		3 (0.7)	
Other		8 (1.9)	
Outcome of most recent TB treatment			
Cured		76 (18.1)	
Completed		158 (37.6)	
Failed category I		109 (26.0)	
Failed category II		11 (2.6)	
Defaulted		45 (10.7)	
Unknown		21 (5.0)	

*Values are no. (%) or no. positive/no. tested (%) unless otherwise indicated. MDR, multidrug resistant; TB, tuberculosis; IQR, interquartile range.

Among PTCs, most had received only 1 previous category I treatment course; 55.7% had a successful outcome (cured or treatment completed) in their last treatment, and 28.6% failed treatment. HIV status was known for 758 (90.2%) patients. Of these patients, 606 were HIV positive (79.9%).

Of 352 NCs tested, 54 (15.3%) had TB strains resistant to >1 first-line drug, and 47 (13.4%) had strains resistant to isoniazid (Table 2). An MDR strain was isolated in 27 NCs, resulting in MDR TB prevalence of 7.7% (95% CI 4.9%–10.5%). Almost half (45.2%) of the 281 PTCs had a TB strain resistant to >1 drug; 127 (45.2%) had a strain resistant to isoniazid, and 95 (33.8%) had MDR TB (95% CI 28.3%–39.3%). Among PTCs, all who had failures of category II treatment (6/6), 83.5% (40/48) who had failures of category I treatment, 23.2% (42/181) relapses, and 13.4% (4/30) who had default treatment were infected with an MDR strain. The proportion of resistance to pyrazinamide among MDR TB strains was 74.1% (20/27) for NCs and 67.4% (64/95) for PTCs.

**Table 2:** Patterns of first-line drug resistance in new and previously treated case-patients with TB, Swaziland 2009–2010*

Resistance pattern	New, n = 352			Previously treated, n = 281	
	No. (%)	95% CI		No. (%)	95% CI
Susceptible to all first-line drugs	298 (84.7)	80.9–88.4		142 (50.5)	44.7–56.4
Any resistance	54 (15.3)	11.6–19.1		139 (49.5)	43.6–55.3
Isoniazid	47 (13.4)	9.8–16.9		127 (45.2)	39.4–51.0
Rifampin	28 (8.0)	5.1–10.8		102 (36.3)	30.7–41.9
Ethambutol	29 (8.2)	5.4–11.1		94 (33.5)	27.9–39.0
Streptomycin	47 (13.4)	9.8–16.9		115 (40.9)	35.2–46.7
Monoresistance	12 (3.4)	1.5–5.3		24 (8.6)	5.3–11.8
Isoniazid	5 (1.4)	0.2–2.7		12 (4.3)	1.9–6.6
Rifampin	1 (0.3)	0.0–0.8		7 (2.5)	0.7–4.3
Ethambutol	0			0	
Streptomycin	6 (1.7)	0.4–3.1		5 (1.8)	0.2–3.3
MDR	27 (7.7)	4.9–10.5		95 (33.8)	28.3–39.3
Isoniazid + rifampin	1 (0.3)	0.0–0.8		3 (1.1)	0.0–2.3
Isoniazid + rifampin + ethambutol	0			2 (0.7)	0.0–1.7
Isoniazid + rifampin + streptomycin	6 (1.7)	0.4–3.1		12 (4.3)	1.9–6.6
Isoniazid + rifampin + streptomycin + ethambutol	20 (5.7)	3.3–8.1		78 (27.8)	22.5–33.0
Other first-line drug-resistance patterns different from MDR	15 (4.3)	2.2–6.4		20 (7.1)	4.1–10.1
Isoniazid + streptomycin	6 (1.7)	0.4–3.1		6 (2.1)	0.4–3.8
Isoniazid + streptomycin + ethambutol	9 (2.6)	0.9–4.2		14 (5.0)	2.4–7.5

*New case-patients were those not previously treated for TB or who had taken anti-TB drugs in the past for <1 month. TB, tuberculosis; MDR, multidrug resistant.

Among strains resistant to isoniazid or rifampin, second-line drug resistance was most frequently seen with ethionamide (Table 3). Of the 10 strains resistant to ofloxacin, 8 were also resistant to moxifloxacin (3 in NCs, 5 in PTCs). Only 1 XDR TB strain was isolated; it was resistant to moxifloxacin.

**Table 3:** Patterns of second-line drug resistance in rifampin- and isoniazid-resistant *Mycobacterium tuberculosis* isolates, Swaziland, 2009–2010*

Resistance pattern	No. (%) isolates resistant to isoniazid or rifampin,† n = 60	No. (%) isolates from case-patients with MDR TB, n = 122
No resistance to second-line drugs	54 (90.0)	72 (49.2)
Any resistance	6 (10.0)	62 (50.8)
Ethionamide	6 (10.0)	58 (47.5)
Ofloxacin	0	10 (8.2)
*p*-aminosalicylic acid	0	0
Cycloserine	0	0
Amikacin	0	2 (1.6)
Capreomycin	0	3 (2.5)
Specific resistance patterns		
Ethionamide	6 (10.0)	50 (41.0)
Ethionamide + ofloxacin	0	6 (4.9)
Ethionamide + amikacin + capreomycin	0	1 (0.8)
Ethionamide + ofloxacin + amikacin + capreomycin	0	1 (0.8)
Ofloxacin	0	3 (2.5)
Capreomycin	0	1 (0.8)
XDR	0	1 (0.8)

*MDR, multidrug resistant; TB, tuberculosis; XDR, extensively drug resistant. †Isolates from MDR TB patients excluded.

In univariate analysis, past TB treatment (PTCs), female sex, HIV infection, and age 28–40 years were significantly associated with MDR TB (Table 4). In multivariate analysis, PTCs and HIV-infected patients were 4× and ≈2× more likely to be infected with an MDR TB strain, respectively, compared with NCs and HIV-noninfected patients. The youngest age group was close to being significantly associated with MDR TB regardless of HIV status and history of previous treatment.

**Table 4:** Patient characteristics associated with risk for MDR TB, Swaziland, 2009–2010*

Variable	No. positive/no. tested (%)	Univariate analysis			Multivariate analysis	
		PR (95% CI)	p value		Adjusted PR (95% CI)	p value
Sex						
M	46/309 (14.9)	Ref			Ref	
F	76/324 (23.5)	1.58 (1.13–2.19)	0.007		1.30 (0.92–1.82)	0.139
Age, y†						
>40	21/156 (13.5)	Ref			Ref	
32–40	41/182 (22.5)	1.67 (1.03–2.71)	0.036		1. 43 (0.90–2.28)	0.132
27–31	27/136 (19.9)	1.47 (0.88–2.49)	0.145		1.34 (0.81–2.23)	0.253
15–26	32/158 (20.3)	1.50 (0.91–2.49)	0.112		1.61 (0.98–2.65)	0.058
Region of residence						
Shiselweni	26/147 (17.7)	Ref				
Manzini	30/169 (17.8)	1.00 (0.62–1.62)	0.988			
HhoHho	27/168 (16.1)	0.91 (0.56–1.48)	0.702			
Lubombo	39/148 (26.4)	1.49 (0.96–2.31)	0.076			
Education						
None	20/139 (14.4)	Ref				
Primary school	45/208 (21.6)	1.50 (0.93–2.43)	0.097			
Secondary school	31/166 (18.7)	1.30 (0.78–2.17)	0.321			
High school	17/89 (19.1)	1.33 (0.74–2.39)	0.346			
Tertiary	5/20 (25.0)	1.74 (0.73–4.11)	0.208			
Employment (permanent job)						
No	83/351 (19.1)	Ref				
Yes	183/37 (20.2)	1.06 (0.74–1.50)	0.753			
HIV status						
Negative	12/114 (10.5)	Ref			Ref	
Positive	102/451 (22.6)	2.15 (1.23–3.77)	0.008		1.78 (1.02–3.10)	0.043
TB patient type						
New case-patient	27/352 (7.7)	Ref			Ref	
Previously treated case-patient	95/281 (33.8)	4.41 (2.96–6.56)	<0.001		4.25 (2.78–6.50)	<0.001

*MDR, multidrug-resistant; TB, tuberculosis; PR, prevalence ratio; Ref, referent. †Age categorized into quartiles.

## Discussion

In Swaziland, 7.7% and 33.8% of TB smear-positive NCs and PTCs, respectively, had MDR. This represents an 8.5-fold and 3.7-fold increase compared with MDR prevalence among NCs and PTCs, respectively, from the previous DST survey in 1995. This prevalence appears to be the highest MDR TB prevalence reported in an African country thus far. In neighboring countries, as in South Africa, the estimated MDR TB prevalence was 1.8% (95% CI 1.5–2.3) in NCs and 6.7% (95% CI 5.5–8.1) in PTCs in 2002 (*5*). In Mozambique, 3.5% (95% CI 2.5–4.7) of NCs and 11.2% (95% CI 4.2–30.0) of PTCs had MDR TB in 2006 (*5*). The next closest value of prevalence of MDR in new case-patients in an African country is nearly half the one observed in Swaziland (Rwanda, 3.9%) (*5*). If we consider the extremely high incidence of TB in the population, the prevalence of resistance observed would result in a high MDR TB population rate, comparable to that observed in former Soviet Union countries. Resistance to fluoroquinolones and second-line anti-TB injectable drugs was surprisingly low, however, with only 1 XDR TB patient seen.

Several factors may have contributed to the increase of MDR TB in Swaziland. First, health services are overwhelmed by a huge increase in TB patients due to the HIV epidemic and a lack of health personnel, which could result in poor TB case management and subsequent development of drug resistance. Second, many citizens from Swaziland regularly cross the border to work in mines in South Africa, where the prevalence of MDR TB is high (*12*). Third, the small size of the country (17,364 km^2^) enables the mobility of the population between regions.

High HIV rates might also play a role. In our study, HIV co-infection was independently associated with MDR TB. The correlation between HIV infection and anti-TB drug resistance remains controversial; there were more frequent associations reported in studies in North America than in studies in Africa (*13**–**15*). Several factors have been proposed to explain such an association. Malabsorption of anti-TB drugs has been documented for HIV-positive patients, which could increase the risk for acquired rifampin resistance (*13*). In settings where HIV infection is linked to socioeconomically vulnerable populations, poor treatment adherence and lack of access to proper treatment may contribute to the development of drug resistance (*14*). Also, persons with HIV/AIDS may be more exposed to patients with MDR TB during hospitalizations or consultations in health structures with insufficient infection control because the complexity of management of MDR TB patients entails frequent visits to health facilities, which also increases the risk for MDR TB nosocomial outbreaks among HIV-infected persons (*16**–**18*). Finally, TB progresses rapidly in HIV-infected patients, who are likely to reactivate an infection acquired recently, compared with HIV-negative patients, who usually reactivate latent infection acquired a long time ago.

Therefore, we could speculate that TB strains harbored by HIV-infected patients are more likely to reflect the strains currently circulating in the community. This suggestion could explain the more frequent association observed between HIV infection and primary MDR TB compared with acquired MDR TB (*14*). In our study, the adjusted prevalence ratio was higher for HIV-infected NCs than PTCs (2.14 versus 1.74), although estimates were not significantly different because of the small sample size (data not shown). We also observed an association between the youngest age group, which is more likely to be recently infected, and MDR TB after correcting for HIV status and history of previous TB treatment. Unfortunately, because of the small sample size, we were not able to further analyze this association by stratified analysis. Nonetheless, the role of HIV infection on the transmission of MDR TB could be further assessed in a cluster analysis of the MTB strains by using DNA fingerprinting data.

In eastern European countries, the specific phylogenetic lineage Beijing *M. tuberculosis* genotype has been identified as a particularly prevalent strain independently associated with MDR TB and transmission, indicating a potential role of this pathogen in the epidemiology of drug resistance in these regions (*19**,**20*). The Beijing strain has also been isolated in several African countries (*21**–**26*), and an association between this strain and the emergence of drug resistance has been reported in South Africa (*27*). Such molecular epidemiologic studies would be recommended in Swaziland.

This study had several limitations. First, the survey population only represented the population of patients diagnosed through the health system. Therefore, not much is known about patients lacking access to health services. The 15 clinics providing TB diagnosis and treatment in the country were included in the survey, but, despite free TB diagnosis and treatment in Swaziland, transport costs and user fees for health facility registration are factors that can limit access to care. Also, almost 16% of the registered smear-positive patients during the survey period were not screened. Although, basic demographic data between assessed and nonassessed patients were not statistically different, we cannot fully exclude the possibility of a selection bias. The proportion of patients with unknown HIV status could have also biased the association seen with MDR prevalence. Therefore, we conducted a sensitivity analysis under a pessimistic assumption that all patients with unknown HIV status were HIV positive and under an optimistic assumption that they were all HIV negative. Figures did not diverge from the original result.

Current WHO recommendations for countries with high anti-TB drug resistance rates and high HIV prevalence are to systematically perform DST at the time of initiation of anti-TB therapy to avoid deaths caused by unrecognized MDR TB (*28**,**29*). The use of rapid drug-resistance testing methods (i.e., PCR) is also recommended to enable a quick start to empirical treatment in potential case-patients while waiting for DST results (*28*). Such strategies require a major effort from national TB programs in terms of laboratory capacity. Line probe assays were recently introduced at the National Reference Laboratory in Swaziland. New technologies, such as the fully automated PCR method of Xpert MTB/RIF (Cepheid, Sunnyvale, CA, USA) would be useful in Swaziland to detect *M. tuberculosis* and resistance to rifampin at lower levels of health care services (*30*).

The national TB program in Swaziland should also consider implementing a surveillance system for anti-TB drug resistance to follow drug resistance trends over time, detect outbreaks in a timely manner, and monitor achievements in infection control and treatment measures (*31**,**32*). Periodic surveys may be a costly alternative in terms of logistics, human resources, and shipment costs (≈€137,000 for this survey). Similarly, the implementation of a treatment strategy able to effectively treat all case-patients with drug-resistant TB in contexts of limited health services capacity, lack of health personnel, and high risk of nosocomial drug resistance transmission is another challenge. The National MDR TB Program of Swaziland was approved in 2009 by the WHO Green Light Committee for a MDR TB pilot project, and organizations such as Médecins Sans Frontières provide support to the program in certain regions. However, the overall response for MDR TB and HIV treatment for co-infected patients still needs to be scaled up.

In conclusion, this study reports a very high prevalence of MDR TB in Swaziland, which currently appears to have the highest prevalence in Africa, and shows a rapid increase in the prevalence of MDR TB in the space of slightly more than a decade. The experience from Swaziland calls for further investigation into the effect of the HIV epidemic on TB drug resistance. These results also highlight the inappropriateness of drug resistance surveys to detect early increase of drug resistance in a country when the surveys are not performed regularly. The lack of recent representative data in many African countries probably underestimates the prevalence of drug-resistant TB in this region (*5**,**33*). The high prevalence of drug resistance in a country already facing a huge epidemic of TB and HIV shows an urgent need for major interventions in terms of detection, treatment, and infection control.

## References

[1] World Health Organization. Global tuberculosis control. Geneva: The Organization; 2010.

[2] World Health Organization. The stop TB strategy: building on and enhancing DOTS to meet the TB related millennium development goals. Geneva: The Organization; 2006.

[3] Badri M, Wilson D, Wood R. Effect of highly active antiretroviral therapy on incidence of tuberculosis in South Africa: a cohort study. Lancet. 2002;359:2059–64. 10.1016/S0140-6736(02)08904-3 12086758

[4] World Health Organization. Guidelines for surveillance of drug resistance in tuberculosis. 4th edition. Geneva: The Organization; 2009.

[5] World Health Organization. Towards universal access to diagnosis and treatment of multidrug-resistant and extensively drug-resistant tuberculosis by 2015. WHO progress report 2011. Geneva: The Organization; 2011.

[6] Joint United Nations Programme on HIV/AIDS. AIDS epidemic update: November 2009. Geneva: The Programme; 2009.

[7] World Health Organization. Global tuberculosis control: epidemiology, strategy, financing. Geneva: The Organization; 2009.

[8] Gandhi NR, Moll A, Sturm AW, Pawinski R, Govender T, Lalloo U, Extensively drug-resistant tuberculosis as a cause of death in patients co-infected with tuberculosis and HIV in a rural area of South Africa. Lancet. 2006;368:1575–80. 10.1016/S0140-6736(06)69573-1 17084757

[9] Ministry of Health and Social Welfare. Report of the rapid survey for the detection of extreme drug resistant tuberculosis in the Kingdom of Swaziland. Swaziland; 2008.

[10] World Health Organization. Treatment of tuberculosis: guidelines. 4th ed. Geneva: The Organization; 2010. 23741786

[11] World Health Organization. 7th meeting of the strategic and technical advisory group for tuberculosis (STAG-TB). Report on conclusions and recommendations. Geneva: The Organization; 2007.

[12] Calver AD, Falmer AA, Murray M, Strauss OJ, Streicher EM, Hanekom M, Emergence of increased resistance and extensively drug-resistant tuberculosis despite treatment adherence, South Africa. Emerg Infect Dis. 2010;16:264–71. 20113557 10.3201/eid1602.090968 PMC2958014

[13] Patel KB, Belmonte R, Crowe HM. Drug malabsorption and resistant tuberculosis in HIV-infected patients. N Engl J Med. 1995;332:336–7. 10.1056/NEJM199502023320518 7816080

[14] Suchindran S, Brouwer ES, Van RA. Is HIV infection a risk factor for multi-drug resistant tuberculosis? A systematic review. PLoS ONE. 2009;4:e5561. 10.1371/journal.pone.0005561 19440304 PMC2680616

[15] Wright A, Zignol M, Van DA, Falzon D, Gerdes SR, Feldman K, Epidemiology of antituberculosis drug resistance 2002–07: an updated analysis of the Global Project on Anti-Tuberculosis Drug Resistance Surveillance. Lancet. 2009;373:1861–73. 10.1016/S0140-6736(09)60331-7 19375159

[16] Centers for Disease Control and Prevention. Nosocomial transmission of multidrug-resistant tuberculosis among HIV-infected persons—Florida and New York, 1988–1991. MMWR Morb Mortal Wkly Rep. 1991;40:585–91. 1870559

[17] Rullán JV, Herrera D, Cano R, Moreno V, Godoy P, Peiro EF, Nosocomial transmission of multidrug-resistant *Mycobacterium tuberculosis* in Spain. Emerg Infect Dis. 1996;2:125–9. 10.3201/eid0202.960208 8903213 PMC2639835

[18] Sacks LV, Pendle S, Orlovic D, Blumberg L, Constantinou C. A comparison of outbreak- and nonoutbreak-related multidrug-resistant tuberculosis among human immunodeficiency virus–infected patients in a South African hospital. Clin Infect Dis. 1999;29:96–101. 10.1086/520189 10433570

[19] Glynn JR, Whiteley J, Bifani PJ, Kremer K. van SD. Worldwide occurrence of Beijing/W strains of *Mycobacterium tuberculosis*: a systematic review. Emerg Infect Dis. 2002;8:843–9. 12141971 10.3201/eid0808.020002 PMC2732522

[20] Pardini M, Niemann S, Varaine F, Iona E, Meacci F, Orru G, Characteristics of drug-resistant tuberculosis in Abkhazia (Georgia), a high-prevalence area in Eastern Europe. Tuberculosis (Edinb). 2009;89:317–24. 10.1016/j.tube.2009.04.002 19539531

[21] Affolabi D, Anyo G, Faihun F, Sanoussi N, Shamputa IC, Rigouts L, First molecular epidemiological study of tuberculosis in Benin. Int J Tuberc Lung Dis. 2009;13:317–22. 19275790

[22] Cowley D, Govender D, February B, Wolfe M, Steyn L, Evans J, Recent and rapid emergence of W-Beijing strains of *Mycobacterium tuberculosis* in Cape Town, South Africa. Clin Infect Dis. 2008;47:1252–9. 10.1086/592575 18834315

[23] Homolka S, Post E, Oberhauser B, George AG, Westman L, Dafae F, High genetic diversity among *Mycobacterium tuberculosis* complex strains from Sierra Leone. BMC Microbiol. 2008;8:103. 10.1186/1471-2180-8-103 18578864 PMC2447842

[24] Kibiki GS, Mulder B, Dolmans WM, de Beer JL, Boeree M, Sam N, *M. tuberculosis* genotypic diversity and drug susceptibility pattern in HIV-infected and non-HIV-infected patients in northern Tanzania. BMC Microbiol. 2007;7:51. 10.1186/1471-2180-7-51 17540031 PMC1913919

[25] Viegas SO, Machado A, Groenheit R, Ghebremichael S, Pennhag A, Gudo PS, Molecular diversity of *Mycobacterium tuberculosis* isolates from patients with pulmonary tuberculosis in Mozambique. BMC Microbiol. 2010;10:195. 10.1186/1471-2180-10-195 20663126 PMC2914001

[26] Groenheit R, Ghebremichael S, Svensson J, Rabna P, Colombatti R, Riccardi F, The Guinea-Bissau family of *Mycobacterium tuberculosis* complex revisited. PLoS ONE. 2011;6:e18601. 10.1371/journal.pone.0018601 21533101 PMC3080393

[27] Johnson R, Warren RM, van der Spuy GD, Gey van Pittius NC, Theron D, Streicher EM, Drug-resistant tuberculosis epidemic in the Western Cape driven by a virulent Beijing genotype strain. Int J Tuberc Lung Dis. 2010;14:119–21. 20003705

[28] Strategic and Technical Advisory Group for Tuberculosis (STAG-TB). Report of the ninth meeting. Geneva: The Organization; 2009.

[29] World Health Organization. Guidelines for the programmatic management of drug-resistant tuberculosis. Emergency update. Geneva: The Organization; 2008.

[30] Boehme CC, Nabeta P, Hillemann D, Nicol MP, Shenai S, Krapp F, Rapid molecular detection of tuberculosis and rifampin resistance. N Engl J Med. 2010;363:1005–15. 10.1056/NEJMoa0907847 20825313 PMC2947799

[31] Cohen T, Colijn C, Finklea B, Wright A, Zignol M, Pym A, Are survey-based estimates of the burden of drug resistant TB too low? Insight from a simulation study. PLoS ONE. 2008;3:e2363. 10.1371/journal.pone.0002363 18523659 PMC2408555

[32] Cohen T, Colijn C, Wright A, Zignol M, Pym A, Murray M. Challenges in estimating the total burden of drug-resistant tuberculosis. Am J Respir Crit Care Med. 2008;177:1302–6. 10.1164/rccm.200801-175PP 18369201 PMC2720088

[33] Ben Amor Y, Nemser B, Singh A, Sankin A, Schluger N. Underreported threat of multidrug-resistant tuberculosis in Africa. Emerg Infect Dis. 2008;14:1345–52. 10.3201/eid1409.061524 18759999 PMC2603092

